# Delivering the unexpected—Information needs for PSA screening from Men's perspective: A qualitative study

**DOI:** 10.1111/hex.13275

**Published:** 2021-06-07

**Authors:** Katrin Kuss, Charles Christian Adarkwah, Miriam Becker, Norbert Donner‐Banzhoff, Kathrin Schloessler

**Affiliations:** ^1^ Department of General Practice/Family Medicine Philipps‐University Marburg Marburg Germany; ^2^ CAPHRI School for Public Health and Primary Care Department of Health Services Research, Maastricht University Maastricht The Netherlands; ^3^ Department of General Practice/Family Medicine Ruhr University Bochum Bochum Germany

**Keywords:** consumer health information, counselling, decision aid, prostate‐specific antigen (PSA), qualitative research, shared decision making

## Abstract

**Background:**

Making decisions about PSA screening tests is challenging, as it requires both knowledge of the possible benefits and harms of screening and an individual assessment of the patient's values. Our research explores how much and what information men perceive to be necessary with regard to screening for prostate cancer.

**Objective:**

To explore men's information and associated needs for decision making in PSA testing.

**Design:**

Qualitative interview study.

**Setting and participants:**

We interviewed 32 men (aged 55‐69) about their decision making on PSA screening following counselling with a Decision Aid at their GP’s or urologist's practice in Germany.

**Main Outcome Measures:**

Men's expressed needs for decision making in PSA testing.

**Methods:**

All interviews were transcribed verbatim and analysed by framework analysis.

**Results:**

Comprehensive pre‐screening counselling is needed. For the men in our study, information about test (in)accuracy, the benefit‐harm balance and consequences of the test were relevant and surprising. Additional needs were for interpretation support, a take‐home summary and time for deliberation. For several men, their physician's attitude was of interest. After being well‐informed, most men felt empowered to make a preference‐based decision on their own.

**Discussion:**

Men were surprised by what they learned, especially regarding the accuracy and possible harms of screening. There is large variation in the breadth and depth of information needed, and some controversy regarding the consequences of testing.

**Conclusion and patient contribution:**

A core set of information should be offered before men make their first PSA screening decision. Information about biopsy and associated side‐effects could follow in a short form, with details only on request. Knowledge about a high rate of false‐positive test results beforehand might help men handle a suspicious test result.

## 1 INTRODUCTION

Until some years ago, and in part to date, cancer screening has been seen almost exclusively positively and uncritically, both in the community^1^, ^2^ and also by many physicians. Early diagnosis of cancer by screening seems to be generally perceived as advantageous, as this allows for early treatment. While the benefits of screening outweigh its possible harms for some types of cancer, this is debatable with regard to screening for prostate cancer (PCa) by testing for prostate‐specific antigen (PSA). PSA screening seems to slightly reduce PCa‐specific mortality, by 1 death per 10.000 person‐years,^3^, ^4^, ^5^ but is associated with psychological or physical consequences. The ethical dilemma of screening is that asymptomatic people are at risk of receiving a diagnosis of cancer which would never have affected them in their life—so‐called overdiagnosis, often associated with overtreatment and corresponding side‐effects.^6^, ^7^

In Germany, PCa screening is common in general practice and ambulant Urology clinics.^8^ However, costs are not covered by the statutory health insurance. Men often undergo the test with a lack of knowledge, in particular with little understanding of the unclear benefit and, furthermore, the potential repercussions of the initially innocuously perceived blood test.^9^ The men tend to an unbalanced perception and overestimated screening benefits.^10^, ^11^ Due to the controversial benefit‐harm balance, many countries around the world have decided against opportunistic mass PSA screening, advocating for an informed, values‐based, individual decision.^12^, ^13^, ^14^ The combination of adequate knowledge and of patients' values comprises the foundation for a ‘good decision’.^15^, ^16^

Such a good decision is difficult to achieve and patients should neither be left to make a decision nor ‘forced’ towards one. Instead, patients should be involved in Shared Decision‐Making (SDM).^6^, ^14^ However, we still observe a lack of SDM in clinical encounters.^17^, ^18^, ^19^ Decision aids (DA) may be used within a consultation to enhance SDM.^20^, ^21^, ^22^ In situations where more than one reasonable option is available, DAs make explicit that a choice exists. They present benefits and harms, optimally supported by pictographs or icons which communicate risk graphically.^20^

Nevertheless, physicians and experts disagree on the amount of information that should be given prior to the screening test. Moreover, men's needs do not necessarily coincide with the presumed needs addressed by physicians. This raises the question of what information men should receive.

One could approach the problem of what a DA should cover by asking ‘the man in the street’ what he would like to know. However, this would, almost by definition, not capture new information of which individuals are not aware. Another approach would be to ask doctors what information should be presented. However, for example, urologists and non‐urologists have different opinions regarding the relevance of various facts.^21^, ^22^ Furthermore, communication goals differ, ranging from convincing men to be screened or *not* to be screened to facilitating and supporting patient choice.^23^ The approach to defining DA content from an objective, scientific point of view is also open to consideration, but does not necessarily coincide with men's needs.

This raises the question of which support should be provided to men.^24^ There are some quantitative studies about PSA testing to determine physicians' rating of the importance of key facts,^21^ to explore which information has an effect on men's interest in screening,^25^, ^26^, ^27^ or to examine their knowledge.^2^, ^11^, ^28^ Also, there are qualitative studies asking patients,^29^, ^30^ GPs,^31^, ^32^ a community jury,^33^ or experts and patients^22^ about PSA screening. These studies were conducted in Australia, the UK and the United States.

Therefore, we aimed to assess the transferability of previous findings to the German setting. Moreover, in exploring the needs of men regarding decision support on PSA screening, needs can imply, in addition to factual information, other decision support.^15^, ^24^ A further focus lies in the degree to which potential (treatment) consequences should be presented obligatorily—for PSA^34^ and for other screening decisions^35^ this is still in debate. Owing to the above‐mentioned problem, we chose a special setting. We interviewed men who had been guided through a structured DA. We aimed to extract practical recommendations to help providers in counselling men adequately and in supporting shared decision making.

## 2 METHODS

### 2.1 Design

The multiphased PSA‐inform project consisted of the development and user evaluation of a transactional DA for PSA screening (*arriba*‐PSA),^36^ followed by a later RCT ^37^ to, among other things, determine the influence of the transactional DA on men's decisional conflict. The results presented here are part of the early qualitative user evaluation after the ODSF‐oriented^15^ development of the transactional DA for PSA screening.^38^ Within this context, we took the opportunity to ask men directly about their own needs regarding clinical counselling. Men were guided by their physician through a sequence of information and deliberation steps following a specified course of talk and graphs irrespective of what they needed to know. Of course, men had the opportunity to ask questions and to obtain additional explanations. The content of the DA, however, was fixed in advance. This provided a unique opportunity to study men's information needs. We assume prior counselling with a DA as prerequisite to evaluating informational needs, as one can hardly assess the salience of knowledge one does not have.

### 2.2 Setting and recruitment

GPs in our research network and urologists in a district of Hesse in Germany were invited to participate in the pilot evaluation of the DA. For counselling, the GPs and urologists attended a two‐hour training session which focused on PSA screening and the application of the consultation with the DA. Afterwards, each physician recruited three to four men, aged 55‐69, representing the core age group of ERSPC,^39^ from their daily consultations. For inclusion, men should have had a preventive check‐up examination or raised the question of PCa screening, respectively. All men had to be asymptomatic regarding the prostate and had to have sufficient German language skills. We included men with and without prior PSA test experience. A sample of 8‐10 physicians and of about 30 men was deemed appropriate to reach diversity in characteristics and to reach thematic saturation.^40^, ^41^ Sample and recruitment considerations were based on the DA development and evaluation study (publication in preparation). Our study was approved by the Ethics Committee of the Faculty of Medicine, University of Marburg (72/13). All participants, men and physicians, gave written informed consent.

### 2.3 Intervention – the DA

The decision aid (*arriba*‐PSA^36^; see Appendix S1) contained information on the decision to be made (no test vs test with a flow chart of potential consequences), basic knowledge about the prostate gland and PSA, epidemiological comparisons of mortality due to PCa in relation to other causes of death, PCa incidence and PCa‐specific mortality of patients who had been screened in comparison with those who had not been screened, test accuracy (false‐positive and false‐negative results), as well as the rate of overdiagnosed PCa. Treatment options were presented obligatorily to about half of the patients; then, during the development process, the list of treatment options moved into the facultative background menu on demand. Benefits (here defined as reduction of PCa‐related morbidity and mortality^6^) and harms (here defined as false‐positive results, psychological harm, complications of prostate biopsy, overdiagnosis, overtreatment, or side‐effects of treatment^6^) were not labelled as ‘benefit’ or ‘harm’ in the DA, as the interpretation has to be made individually. Accordingly, the DA ends with a summary and a handout with a short value clarification exercise.

### 2.4 Data collection and analysis

Individual, semi‐structured face‐to‐face interviews with men as key informants were conducted by the study team (KK, KS, MB, CCA) between 1‐2 weeks after the men had received counselling with the DA. This time span was chosen to allow men recollection of the DA content for the interview. If necessary, the DA slides were shown again but not discussed within the interview to check for comprehension. All interviewers are experienced health professionals and researchers and participated in a shared training of the interview guide (extract see Appendix S2). All interviews were audiotaped, pseudonymized and transcribed verbatim.

We used the Ottawa Decision Support Framework (ODSF) to inform the guideline and to map our findings. Following the ODSF, we clustered the needs of men in three domains (gaining knowledge, getting support and becoming aware of own preferences).^15^, ^16^ All interviews, originally focused on evaluating the DA, were fully coded. Afterwards, all interviews were recoded and re‐analysed with regard to the focus of information needs. Only those statements which were given directly as an answer to a question that focused on decisional needs or which were markedly accentuated by the interviewee, for example by adjectives or frequent iterations, were eligible for this secondary analysis. We derived the first coding scheme deductively, based on the interview guide and the ODSF, and augmented it inductively according to emerging themes.

Our analysis was based on the framework approach.^42^, ^43^ Specifically, we sorted recurring or important themes into a category system (coding scheme), built thematic and central charts and abstracted them for overall analysis and interpretation. The coding and analysis were led by KK, who coded all interviews, assisted by KS, CCA, and MB, who each coded four interviews independently to check for validity and reliability. Discussion of findings and interpretations within the research team helped to ensure credibility and trustworthiness. The analysis was assisted by MAXQDA software for qualitative data analysis, version 12.^44^

## 3 RESULTS

### 3.1 Participants

Nine physicians (7 GPs, 2 urologists) and 32 men participated in the study. Due to the technical loss of one interview, we analysed the interviews of 31 men. The average interview length was 29 minutes (range 8‐56 minutes; SD 12.2; IQR 17.6). Half of the men reported having had a prior PSA test. The characteristics of study participants are summarized in Table 1 and Table 2, respectively.

**TABLE 1:** Characteristics of men

	Analysed (n = 31)	Counselled (n = 32)
Age; mean (SD; range)	61.4 (4.1; 55‐69)	61.2 (4.1; 55‐69)
Education (highest degree); n (%)		
University degree	13 (41.9)	13 (40.6)
High school (12‐13 y)	4 (12.9)	4 (12.5)
Secondary school	4 (12.9)	5 (15.6)
Lower secondary school	10 (32.3)	10 (31.3)
Marital status; n (%)		
Married	25 (80.6)	25 (78.1)
Unmarried	1 (3.2)	2 (6.3)
Divorced	5 (16.1)	5 (15.6)
Previous PSA tests; n (%)		
PSA test done ≥1 time	15 (48.4)	15 (46.9)
PSA test NOT done	7 (22.6)	7 (21.9)
Missing	9 (29.0)	10 (31.3)
PSA test decision after counselling; n (%)		
PSA test	13 (41.9)	14 (43.8)
No PSA test	10 (32.3)	10 (31.3)
Not decided yet	3 (9.7)	3 (9.4)
Missing	5 (16.1)	5 (15.6)

Abbreviations: DRE, digital rectal exam; PSA, prostate‐specific antigen.

**TABLE 2:** Characteristics of physicians

	Counselling (n = 9)
Age; mean (SD; range)	50 (10.1; 28‐62)
Sex; n (%)	
Male	6 (66.7)
Female	3 (33.3)
Specialty; n (%)	
General practice	6 (66.7)
Internal medicine	1 (11.1)
Urology	2 (22.2)
Practice setting, n (%)	
City (20.000‐100.000)	4 (44.4)
Town (5.000‐20.000)	3 (33.3)
Small town (<5.000)	1 (11.1)
Missing	1 (11.1)
Previous experience with the DA, other modules, only GP; n (%)	
Yes	7 (100.0)
Regular use thereof	3 (42.9)

### 3.2 Decision‐making needs and decision support

Although nearly half of the men had already had one or more PSA tests before, they were surprised by what they heard and learned in the specific setting of this study.

#### 3.2.1 Clarify decision and needs—Need for pre‐screening counselling

One of the main results is that the men stated that they aim to be thoroughly informed. For them, it should be made clear that they have options (to test—not test/postpone decision) and that an individual decision should be based on their own values and preferences. Several men noted that they either had not been informed before having a PSA test or that they lacked comprehensive and neutral information. In contrast, two men stated that for them the test result is the only relevant information. They would not need any information on test characteristics. After participating in this study, two men felt that their doctors should have been more knowledgeable regarding PSA testing and its consequences.

‘to see this numerically now, statistically in comparison. Some things surprised me then’(0601; man)

‘The pros and cons. So that I can choose. Otherwise, I don’t have a choice’. (0604; man)

#### 3.2.2 Knowledge and expectations—information needs

Information needs were discussed according the structure of the DA: information about the prostate and PCa, test (in)accuracy, benefit‐harm balance, possible consequences of the PSA test and of overdiagnosis/overtreatment. With regard to facts men ought to know, we identified several consistent key themes. However, men differed regarding the information they found most important for their decision making. Nevertheless, we identified three issues important for most men: the test (in)accuracy, the benefit‐harm balance and awareness of subsequent procedures/consequences. The amount of overdiagnosis was seen as essential by some patients, while test consequences were seen controversially. All of this information also took men by surprise. Table 3 summarizes men's needs (✓) and controversial needs (contr.) and compares them with the recommendation and the DA used.

**TABLE 3:** Comparison of informational needs according to ODS‐Framework, the information provided (DA) and informational needs stated by counselled and interviewed men

Different informational needs	ODSF	DA used	Men' needs
Knowledge and expectations about PCa	✓	✓	✓
Options (PSA test; no PSA test)	✓	✓	✓
Natural course of the disease	✓	✓	✓
Benefit‐harm balance	✓	✓	✓
Overdiagnosis^a^	✓	✓	Contr.
Test accuracy	✓	✓	✓
Consequences			
In detail (e.g., biopsy)	✓	✓	Contr.
Awareness of Consequences	✓	✓	✓
Treatment options^b^	(✓)	(✓)	X

Note

✓: information recommended (ODSF/DA) or needed (men); X: information not recommended (ODSF/DA) or not needed (men); (✓): presented only to a sub‐sample, then removed from the DA.

Abbreviation: Contr.: contrary views of interviewed men.

^a^ Overdiagnosis is perceived as part of the benefit/harm balance for Frameworks, while it was rated controversial by patients

^b^ Treatment options were moved into the background text of the DA in the course of the development‐evaluation process. In Frameworks, they are perceived as part of the benefit/harm ratio. However, they are not always listed as a separate need.

During the interviews, we learned that the men had little prior knowledge of the prostate, PCa and PCa screening. Nearly all of the interviewees perceived information on incidence and mortality as helpful in understanding that PCa is a common condition in older men, but the mortality is quite low. However, there was a tendency to mistake the incidence and mortality of PCa.

The most impressive theme, mentioned by men as crucial to a decision for or against a PSA test, was the chance and dimension of inaccurate test results. Men were surprised by the high rate of false‐positive as well as false‐negative test results. Especially the consequences of a false‐positive test result made men consider whether to take the risk of becoming worried.

*‘Um, the risk, um, that you get a positive result, which eventually after all isn’t positive, that is crucial to me. Um, well, […] that there also occur misjudgements relating to this test, and that […] strains my nerves too much. Then better do nothing’. (0102; man)*

In an important finding to the contrary, some men stated that the knowledge and magnitude of false‐positive test results empower them to handle an abnormal test result. They learned that a suspicious result would not be tantamount to a definite diagnosis of cancer.

‘[…] that of course you can also say for now: maybe this was a false positive result? That’s […] an important guidance! […] also for the time being calming’. (0603; man)

While the benefit of screening was rated as important information, almost no man explicitly mentioned the term ‘harm’ during the interviews. In general, men emphasized the usefulness of the comparison of being tested or not as a basis for balancing the pros and cons individually. Men were surprised by the very small effect of one prevented death in a thousand men in 10 years. Interestingly, they came to varying conclusions. While some perceived the benefit as disillusioning, others felt that testing was worth it to save this life.

*‘[…] the key element for me, which is just in my mind or strong within my recollection, the one person, who is surviving in the end […] has the chance, to survive. That was just decisive to me’. (0201; man)*

*‘Well! That the likelihood, that […] brings a sensible cure, which is life‐saving, that this is greatly small. And all these matter ahead, that this is accompanied by very, very much, well, rumination, strain, psychic strain […] decision‐making, which is not necessarily in an adequate proportion to which result is obtained. Well, thousand‐to‐one, that’s a result, but: Is it worth this?’ (0301; man)*

The most controversy observed concerned whether information regarding potentially following the next stages after screening—the ‘string of consequences’—should be presented inherently as part of the counselling or not. However, there was controversy about the degree of details regarding subsequent tests (biopsy), treatments and corresponding side‐effects given at this stage. Several men were not aware of the implication that the initial decision could initiate further action in case of a suspicious test result. Having this knowledge beforehand could influence decision making.

While from one‐third of the men there was no clear statement on the degree of information needed, the other two‐thirds held widely differing views. Nearly half of them stated that potential further stages and consequences should always be mentioned as basic information. However, the other half thought that further detail is only necessary in case of a suspicious PSA test result. For them, information should be given step by step and not in advance. These results were irrespective of having seen the consequences in detail (half of the men) or only as a rough scheme.

‘It always is arguable, ‘what are the consequences’? That’s just the problem, these procedures may be more harmful than what they remedy, and thus I want to know about the ins and outs in advance, yes’ (0301; man)

‘That doesn’t have to be discussed in detail for the moment. […] I consider that you don’t have to look ahead three, four steps like in a chess game’. (0601; man)

The concepts of overdiagnosis and overtreatment were completely new for most men. Living onwards with a known diagnosis was felt to be very burdensome. In a few men this information was the crucial point for their decision making.

#### 3.2.3 Support and resources—additional needs

**FIGURE 1:**
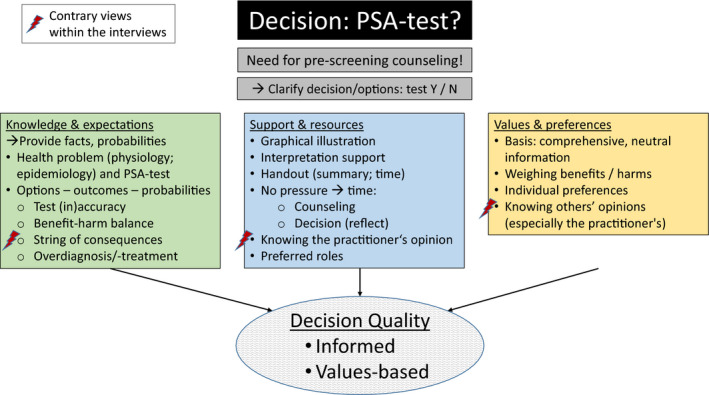
Men's decision support needs

In addition to facts about the PSA test itself, men highlighted further needs regarding support for decision making (see Figure 1).

With regard to the specific setting of counselling on the basis of a DA, men rated the pictographic illustrations (affected men per thousand) a great assistance in terms of comprehending facts and statistics. Beyond that, several men deemed interpretation of the statistics and the possibility to check understanding during a shared consultation essential.

Men welcomed the introduction of a handout to take home. This should primarily recapitulate the facts in the same way as in the counselling. After its introduction, men rated it as a memory aid and an opportunity to reconsider the variety of—for the predominant part of participants—new and surprising information and the in part contradictory facts calmly at home.

A further, frequently expressed need was related to the process and timing of decision making. Men demand time from the provider for comprehensive counselling and to follow and to ‘digest’ the complex information. They were thankful that they had not been pressed for an immediate decision, as many need time to consider the information and to weigh up the pros and cons prudently. Before the handout was introduced, a man said:

*‘But […] for me as a patient it would be important to have all this in written form and then to have a look‐see myself. Calmly. […] I guess, when sitting at a doctor, you will be overloaded, of course. So, you sit there, you gather things and then I guess, […] only at home I really begin to think about it’. (0102; man)*

Regarding decision making, the men's opinions varied on whether they wanted to know the physician's preference regarding PSA screening. Several men were eager to know their doctor's recommendation, which was regarded as highly relevant for their own decision. In contrast, others wanted to hear neither this potentially influencing opinion nor a prescription, like ‘you have to do it’:

‘I go to the doctor and have all what he deems appropriate or necessary'. (0502; man)

‘[…] just an opinion is not what I need to form my own view’. (0604; man)

Most men allow or even seek a physician's statement, but want to make the final decision themselves. Several men said that up to now they had done whatever their physician said, but after having all the new information they felt empowered to make the decision themselves or together with the physician. Two men who had participated in the PSA test for several years suggested giving comprehensive information and time for consideration, especially for first‐time decision making:

‘I could only wonder: why didn’t the counseling take place five years ago, before I did it [the PSA‐test] the first time?’ (0502; man)

#### 3.2.4 Values/preferences

To realize their own values and, for an individual, make a values‐based decision, men noted that comprehensive, neutral information was an essential precondition. Consequently, men felt that no information should be left out. Otherwise the presentation would be biased/not neutral. In addition to individual preferences, knowing others’ opinion, especially the physician's, was mentioned by several men as helpful in order to better understand their own values.

## 4 DISCUSSION AND CONCLUSION

### 4.1 Discussion

Within this study, we used the unique opportunity to explore PSA screening information and further needs of men who were counselled with a structured DA. First of all, the results revealed a large variation regarding the breadth and depth of information men need to know. Moreover, in the specific setting of this study, men were highly surprised by what they learned. While our findings mostly correlate with those of previous studies,^21^, ^29^, ^33^, ^45^, ^46^ we examined the transferability for the German setting.

Our study provides further evidence for a general need in men for comprehensive, balanced information and a clarification that an individual, values‐ and preference‐based decision is to be made. In particular, this needs to take place before the decision is made the first time. Having all the relevant knowledge empowers men to take responsibility and to make an autonomous informed decision or to substantially participate in shared decision making.^47^

Regarding knowledge and expectations, the most impressive themes were the high rate of false‐positive test results, the screening benefit (negligible versus worth doing) and the awareness of potential screening harms. All of this information and also the concept of overdiagnosis took men entirely by surprise; this information was completely new to them. Interestingly, in our study the knowledge of the high rate of false‐positive test results beforehand had a calming effect regarding a possible suspicious test result. A potential explanation could be that this was a hypothetic situation due to the absence of actual suspicious test results in our sample. Whether this reassurance would persist over following steps is debatable. Thus, men in a cohort study who had had a suspicious screening result followed by a benign prostate biopsy result reported negative psychological effects such as substantial thinking and worrying about prostate cancer.^48^ One should keep in mind that biopsies also can cause physical distress.^49^

While men agree on the necessity of nearly all the themes mentioned above, albeit with individual significance, there are differing views regarding two themes (test accuracy and overdiagnosis). This includes the amount of detail on the consequences of a suspicious PSA test. Nevertheless, giving short information on potential consequences and side‐effects before testing could influence the screening decision.^46^ Several men expressed a demand for such information, although there was a tendency to make the decision step by step. Other studies support our findings.^33^, ^50^, ^51^

Within our results, two further aspects are worth mentioning. First, designating a test result as ‘positive’ or ‘negative’ could be misinterpreted by men; a less ambiguous wording would be ‘suspicious’ and ‘unsuspicious’, respectively. A second source for misunderstanding was identified regarding the interpretation of incidence and mortality.

In addition to factual information, further support‐related needs were mentioned. Physicians were seen as the preferred source of information and were expected to interpret available information. Particularly for previously uninformed men, the amount of new, surprising and conflicting information could lead to increased uncertainty and therefore could arouse decisional conflict. To make up their mind, men asked for information materials they could take home in order to digest what they had learned; for the same reason, they felt they needed some time to consider the information and not feel pressured to make an immediate decision. These results correspond with those of other studies.^29^, ^33^, ^50^, ^52^, ^53^

Another area of disagreement was found regarding the wish to hear the physician's attitude. Many, but not all men, were interested in this. The reason could be age‐related, that is in the generation of men aged 55 years and above, many wanted to know their physician's opinion; younger men are potentially more informed themselves or more ready to engage in SDM. Moreover, doing ‘a simple blood‐test’ could have been perceived as not representing a real decision. As it is a routine test, it was not considered inconvenient, harmful or to induce consequences.^53^ Following one's doctor's advice without performing an information search or deliberating is a means of reducing cognitive overload due to the amount and complexity of information and of avoiding ambiguity. Nevertheless, after getting all the information, most men felt empowered to make the decision on their own or in SDM.

The general question remains how to prepare men with adequate information, if any, in primary care. There are ethical/practical polarities and at least three positions one could take. First, if a person does not raise the PSA screening issue himself, one could argue that not providing information to this person is ‘protecting’ him from making a difficult, complex decision or from potential harm due to screening consequences. However, letting men go uninformed (by the GP) has two sides. On the one hand, men will not be confronted with the choice of taking a test which might possibly be more harmful than beneficial. On the other hand, men may receive information, selective and biased, from other sources. Second, and on the other hand, one could argue for supporting men's autonomy by giving all men complete and equal information. However, with regard to providing a large amount of complex information, this would imply supporting the person in value clarification and decision making. Nevertheless, with regard to ‘nudging’, this approach involves the danger of attracting attention towards the screening test.^54^

Third, our data and that of others show^22^, ^24^, ^46^ that the high variability of needs seems to invite a highly individualized approach to counselling. However, the fact that the men in our study rated some of the information obtained as both surprising and highly important does suggest a different procedure. Adjusting the discussion to individually stated wishes should not go too far. There is always the danger that blind spots or misconceptions persist. Therefore, the counselling or a DA must contain a core set of information which is presented to every person who undergoes counselling. In the case of PSA testing, this should include a short description of the relevant anatomy/epidemiology as a basis of what we are talking about, especially as the prostate is a hidden organ and is sometimes tainted with shame.^55^ Further core issues are options (test–no test), test accuracy, benefits and the full spectrum of harms,^53^ disclosing potential consequences and addressing men's own values and preferences. We based our core set on men's surprise at unexpected facts in tandem with the importance they placed on this information. However, we acknowledge that the amount of information is still on debate.

Some essential information is necessary in order to understand the issues at hand, possible options and their implications. Just as the term ‘shared decision‐making’ does not mean completely leaving the decision to the consumer patient, we think a component of paternalism (providing a core set of information) is necessary to support patients in making good decisions. However, this information should be discussed and not only ‘delivered’.^56^, ^57^ We think this has implications for all DAs irrespective of the clinical problem addressed here.

In addition to these three positions, some authors have already suggested an absolute minimum of information, for example for people with little interest or with low health literacy. If approached by a man who may be eligible for screening, at least offer to provide information and in particular make clear that a decision is to be made, that there are different options, that screening can be both helpful and harmful, and that declining is possible.^20^, ^46^ Further information can be given if needed or wished.

Providing physicians with adequate information is a prerequisite for ensuring objective, comprehensible counselling. This is best done by DAs, which can increase the knowledge and awareness about testing and empower men to make a decision.^46^ Moreover, as not all physicians who are consulted by men for a PSA test are sufficiently informed on their own,^58^, ^59^, ^60^ and a great proportion of physicians seem not to apply guidelines correspondingly,^33^, ^50^ DAs may thus improve the counselling provided by physicians.^23^, ^61^

### 4.2 Strengths and weaknesses

This study provides a valuable insight into screening information needs as a prerequisite to informed decision making for asymptomatic men. Our analysis regarding information needs was deductive, and further issues were also derived inductively. Although the DA‐based counselling could concurrently have biased men's felt needs, they would not have reflected on all aspects if they had not been confronted with them previously. Sometimes it was difficult to distinguish whether issues were just very surprising or genuinely essential. For this reason, we re‐analysed all interviews, focusing on the study aims, recoded the interviews independently and discussed the findings critically. Further limitations concern the physicians' dedicated recruitment of participants, which led to the situation that several participants had originally visited their physicians for reasons other than a PSA screening. Although our sample of 32 men is not representative, the sample of men reflects diversity with regard to educational status, previous PSA test experience and test decision after counselling, as well as to several attitudes identified in the interviews. We chose a special setting for our purposeful sampling and reached thematic saturation within the interviews. Our results contribute to the on‐going discussion which core set of information should be given prior to screening examinations, but should be validated by a larger sample size.

### 4.3 Conclusion

Men were highly interested and grateful for the education they received by structured counselling on the basis of a DA. Regarding information, they agree on the absolute necessity of most themes. Nevertheless, we found large variation regarding the breadth and depth of information needs. Many of the issues broached caused surprise; at least, cursory information on test accuracy and unknown constructs such as overdiagnosis were considered necessary. In addition to information, other needs became apparent, such as interpretation support, an information leaflet to enable reflecting on the information and time for making the decision. In any case, merely a recommendation or having the test without any or only selective, biased information is not what men expected.

### 4.4 Practice implications

Even if men had undergone screening tests several times, their baseline knowledge differed, and many were not aware that these tests can cause at least some harm. After consultation with a Decision Aid, unbiased, newly impressive and in part surprising information arose. This calls for a core set of information which should be presented to each man. Optimally, this should be done before a man's first screening decision, as we know that the strongest predictor for having a PSA test is having previously done PSA tests.^46^ The presentation should include information about options, benefits and harms, including overdiagnosis, uncertainty and that further steps and associated side‐effects could follow in the event of a suspicious test result in short form. The degree of detail, but not the core components, could be adapted with regard to interest, knowledge and intellect, but should not be anticipated subjectively beforehand. This semi‐individualized approach is not completely standardizable, but rather underpins the need for personal counselling, paying attention to a man's reaction to the core information provided. Advance knowledge about the possibility of false‐positive test results puts a suspicious test result into perspective.

## CONFLICT OF INTEREST

KK, KS, CCA and MB have no conflicts of interest to declare. NDB is Co‐CEO of the ‘Gesellschaft für Patientenzentrierte Kommunikation’ (Organization for Patient‐centred Communication). This is a registered non‐profit entity contracting with health insurers and providers aiming at the dissemination of *arriba* decision support software. He obtains no financial revenue from this organization apart from travel expenses.

## AUTHOR CONTRIBUTIONS

NDB designed the study, KK, KS, CCA and MB participated in the acquisition and analysis of data. All authors discussed and interpreted the data and the results. KK drafted the manuscript; KS, CCA, MB and NDB revised it critically; and all authors have approved the final article.

## INFORMED CONSENT AND PATIENT DETAILS

We confirm that all patient/personal identifiers have been removed or disguised so the patient/person(s) described are not identifiable and cannot be identified through the details of the story.

## Supporting information

Appendix S1

Appendix S2

## Data Availability

The data that support the findings of this study are available from the corresponding author upon reasonable request.
