# Supplementary material for: Delivering the unexpected—Information needs for PSA screening from Men's perspective: A qualitative study
Source: Health Expect. 2021 Jun 7;24(4):1403–12. doi: 10.1111/hex.13275 (PMC8369103; doi:10.1111/hex.13275)
Supplement: Supplementary file 1 — Appendix S1 [file HEX-24-1403-s001.docx]

**Appendix S1: The Decision Aid arriba –PSA**


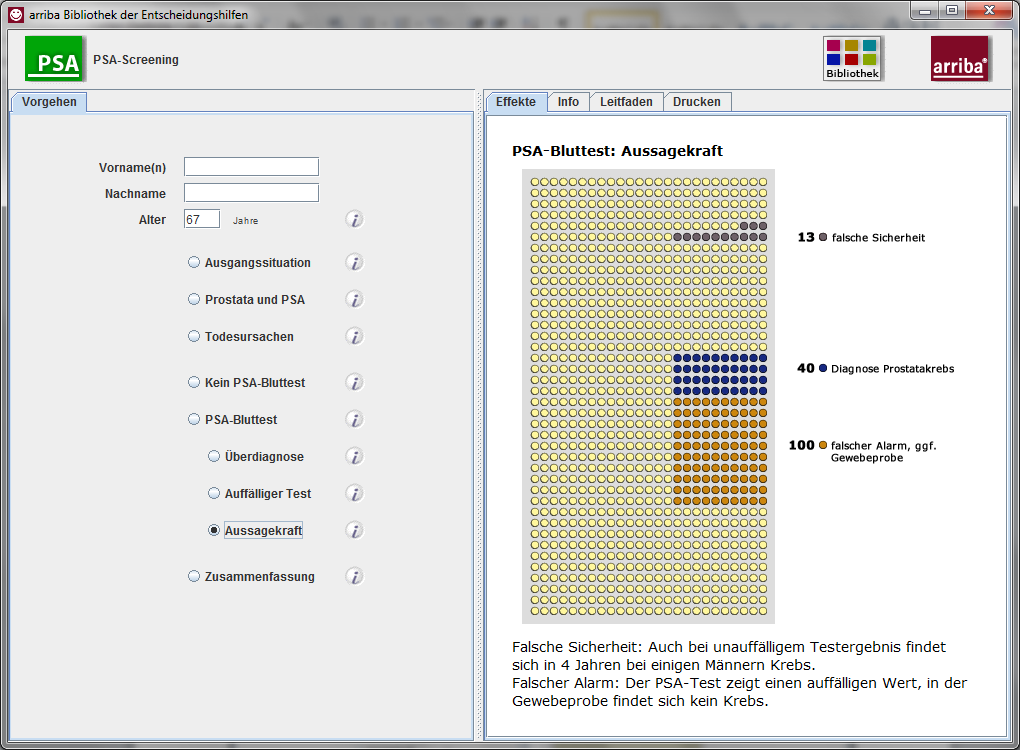


**Figure 1: Screenshots of the Decision Aid arriba-PSA on test accuracy**

*Test accuracy presents abnormal results in total (n=140) divided in false positive results (n=100) and true positive results (n=40). However, false negative results according to a PSA-threshold of 4ng/ml occur in 13 cases.*


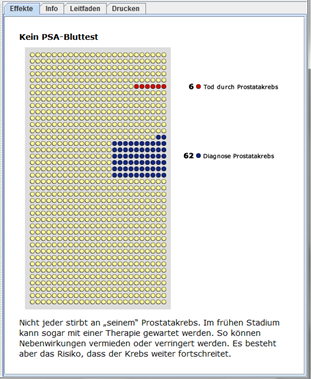

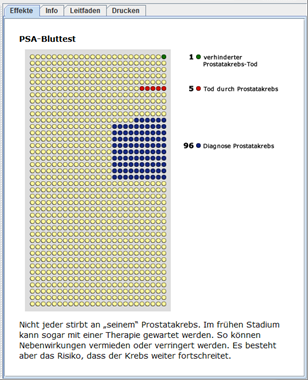


**Figure 2: Comparison of PSA-Test and no-PSA-Test (Diagnosis and Prostate Cancer specific Mortality)**

*The pictograms represent mortality (red) as well as diagnosis of prostate cancer (blue) when men decide against (left) or for (right) having a PSA-test. The difference in prostate cancer specific mortality is highlighted in green. The explaining text states that not everyone dies of “his” prostate cancer. In an early stage, one can even wait with therapy. By doing so, side effects of procedures might be avoided or reduced. However, there is a risk, that prostate cancer might increase/progresses.*
