# Supplementary material for: Delivering the unexpected—Information needs for PSA screening from Men's perspective: A qualitative study
Source: Health Expect. 2021 Jun 7;24(4):1403–12. doi: 10.1111/hex.13275 (PMC8369103; doi:10.1111/hex.13275)
Supplement: Supplementary file 2 — Appendix S2 [file HEX-24-1403-s002.docx]

**Appendix S2**

**Supplement 2: Interview guide – Extract of relevant themes (men’s interviews)**

| - Which experiences do you have with screening in general? Did you already have a prostate-cancer screening? |
| --- |
| - Which information do you consider of importance? What do you need for your decision? |
| - How easy to understand did you experience the counseling with the PC-program? How do you rate the graphic design/the graphics? |
| - Retrospectively – what was most important in making your decision? |
| - In one sentence, please summarize the most important message to you. |
| - What did you miss in the DA? What could be left out? |
| - What of the DA was of advantage to you? (information new, helpful, reluctant, trusthworthy, sceptical) |
| - Any more suggestions/criticism/themes? |
